# Temporal relationships among changes in the RR‐interval and the powers of the low‐ and high‐frequency components of heart rate variability in normal subjects

**DOI:** 10.14814/phy2.15557

**Published:** 2023-01-26

**Authors:** Yuji Yokobori, Hayato Nakane, Chirika Uehara, Toru Nagasawa, Satoshi Mitsuyama, Kiyomi Ohkawa, Kazuomi Kario, Seiji Ozawa

**Affiliations:** ^1^ Department of Healthcare Informatics Takasaki University of Health and Welfare Takasaki Gunma Japan; ^2^ Division of Cardiovascular Medicine, Department of Medicine Jichi Medical University School of Medicine Shimotsuke Tochigi Japan

**Keywords:** heart rate variability, high‐frequency component, low‐frequency component, ultra‐short‐term spectral analysis, vagal nerve activity

## Abstract

Spectral analysis of heart rate variability (HRV) is widely used as a non‐invasive method to assess the cardiovascular autonomic function. Of the two main frequency components of HRV, namely low‐frequency (LF, 0.04–0.15 Hz) and high‐frequency (HF, 0.15–0.4 Hz) components, it is generally accepted that the HF power reflects modulation of heart rate which is mediated by cardiac parasympathetic (vagal) nerve activity. In contrast, the origin and functional correlates of the LF component are still controversial. Although several lines of evidence have indicated a close correlation between LF power and the baroreflex modulation of autonomic outflows, the detailed mechanisms underlying the genesis of the LF component remain unclarified. In this study, we conducted an ultra‐short‐term (UST) spectral analysis of R‐R interval (RRI) time series using Fast Fourier Transform (FFT) with 5‐ and 25‐s windows to clarify the temporal relationships among transient changes in the RRI and, LF and HF powers in healthy subjects. We found that during active standing, transient RRI increases occurred sporadically. The UST spectral analysis revealed that this RRI increase was associated with a simultaneous increase in HF power which was closely linked to the prominent LF power increase. These results indicate that during active standing, increases in LF and HF powers occur simultaneously, and they may reflect enhanced cardiac vagal activity which generates transient bradycardia.

## Graphical abstract

Through ultra‐short‐term spectral analysis of heart rate variability (HRV) in healthy subjects, we found, during active standing, a simultaneous transient increase in the high‐frequency (HF) power of HRV with the onset of transient increase in the RR‐interval (transient bradycardia). This HF power increase was linked to a prominent increase in the low‐frequency (LF) power. The results indicate that the increases in LF and HF powers may reflect enhanced cardiac vagal nerve activity that generates the transient bradycardia.

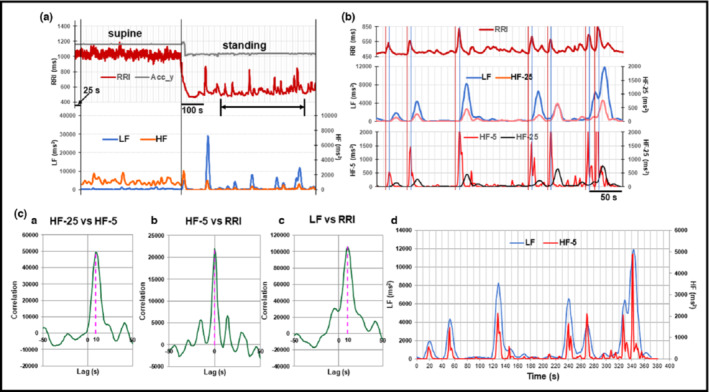

## 1 INTRODUCTION

Heart rate variability (HRV) is the variability in the time between successive heart beats, controlled by the cardiac sympathetic and vagal (parasympathetic) activities (Billman, 2011; Task Force of the European Society of Cardiology and the North American Society of Pacing and Electrophysiology, 1996). Akselrod et al. (1981) reported the use of HRV power spectral analysis to quantify autonomic nervous system cardiovascular control, which provided basic information on spectral power distribution as a function of frequency.

In the spectrum calculated from a beat‐to‐beat time series of the R‐R interval (RRI), three main components are distinguished: very‐low‐frequency (VLF; 0.003–0.04 Hz), low‐frequency (LF; 0.04–0.15 Hz), and high‐frequency (HF; 0.15–0.4 Hz) components. Although the physiological correlates of the VLF component are largely unknown, the powers of the LF and HF components have been considered to reflect autonomic nervous system activities (Billman, 2011; Task Force of the European Society of Cardiology and the North American Society of Pacing and Electrophysiology, 1996).

It is generally accepted that the HF power reflects modulation of heart rate mediated by parasympathetic cholinergic nerve activity (Akselrod et al., 1981; Eckberg, 1997; Hayano et al., 1991; Malliani et al., 1991), whereas the origin and functional correlate of the LF component are controversial. The LF power has often been assumed to provide an index of cardiac sympathetic activity. Malliani et al. proposed that increased sympathetic activity is characterized by an LF to HF power ratio (LF/HF) in favor of the LF component, whereas the opposite occurs during an increase in vagal nerve activity, a notion referred to as sympathovagal balance (Malliani et al., 1991; Pagani et al., 1986). However, accumulating evidence has revealed that this greatly oversimplifies the complex interaction between the sympathetic and parasympathetic divisions of the autonomic nervous system (Billman, 2013; Eckberg, 1997; Goldstein et al., 2011; Hayano & Yuda, 2019; Houle & Billman, 1999).

It is proposed that LF oscillations in the HRV are mediated by vagal activity oscillations driven by the baroreceptor reflex (Cevese et al., 2001; Grasso et al., 1997). This is well supported by the finding that large‐dose atropine, a parasympathetic blocker, abolished almost all spectral powers in the LF and HF ranges (Eckberg, 1997; Jokkel et al., 1995; Koh et al., 1994; Pomeranz et al., 1985). Furthermore, Goldstein et al. examined the relationship among LF power, cardiac sympathetic innervation, and baroreflex function in patients with chronic autonomic failure, and concluded that LF power reflects baroreflex modulation rather than cardiac sympathetic tone (Goldstein et al., 2011; Moak et al., 2007; Rahman et al., 2011). These authors demonstrated a correlation between the LF power and baroreflex‐cardiovagal gain, and that patients with neurogenic orthostatic hypotension and baroreflex‐cardiovagal failure have very low LF power.

In the present study, we attempted to clarify the temporal relationships among transient changes in RRI and the LF and HF powers of normal subjects over the supine to standing postural change, as this is key to elucidating the origin and functional correlates of LF power. To this end, we conducted an ultra‐short‐term (UST) spectral analysis of R‐R interval (RRI) time series using Fast Fourier Transform (FFT) with 5‐ and 25‐s time windows. It has been pointed out that there are restrictions on the use of UST analysis as a proxy for the widely used short‐term (~5 min) and long‐term (≥24‐h) analyses to obtain frequency‐domain parameters for clinical medicine and personal health care (Burma et al., 2021; Shaffer et al., 2020). However, we found that the UST spectral analysis used in the present study was very effective for assessing the dynamics of the RRI sequence and transient changes in the LF and HF spectral components of HRV.

## 2 METHODS

### 2.1 Subjects

Twenty volunteers (10 males and 10 females, mean age 21.3 ± 0.7 years old) participated in this study. They were in good health without cardiovascular and respiratory diseases, and were not taking presubscription medications regularly. All participants gave their informed consent to the study protocol, which was approved by the Ethical Committee of Takasaki University of Health and Welfare.

### 2.2 Experimental protocols

We examined the effects of postural change from supine rest to active standing. Subjects wearing the devices described below adopted a supine position for 15 min, then stood up quietly, and remained standing for 15 min. They were asked to do this 3 times a day between 8:00 and 18:00.

In preparatory experiments, we measured the respiratory frequency of the subjects. In both supine and standing positions, it ranged between 14 and 18 breaths/min (bpm). The mean values were 15.5 ± 1.0 bpm in the supine, and 16.1 ± 1.4 bpm during standing (mean ± SD, *n* = 20). As these values were higher than 9 bpm (=0.15 Hz, the HF component lower frequency limit), we did not control the respiratory frequency in the present study.

### 2.3 Instrumentation

The HRV was measured using a wearable electrocardiogram sensing device (myBeat; Union Tool, Co., Tokyo, Japan). This device was worn around the epigastrium on the chest of the subject, and detected the peak of the QRS complex to obtain beat‐to‐beat time series of the RRI. A three‐axis accelerometer is also incorporated in this device. As the *y*‐axis coincided with the vertical axis of the subject's body, it was useful for detecting the time point and duration of the postural change between supine rest and standing.

We occasionally evaluated the regularity of respiratory frequency and depth during the HRV measurement using a respiration monitor device (ResMo; Aimedic MMT, Tokyo, Japan) that enables to detect thorax volume change using a thoracic belt incorporating a pair of stretchable strain sensors.

### 2.4 Data processing and analyses

To process and analyze the time series of the RRI we used the RRI Analyzer 2 software program (Union Tool, Co.).

As the RRI time series occasionally contained abnormal values caused by noises, the RRI value that deviated from the range between 300 and 2000 ms was omitted. In addition, if the instantaneous heart rate was either 30 bpm greater or less than the average of the preceding 8 values, it was also omitted. Continuous RRI signal data as a function of time was obtained from discrete RRI event series using a spline interpolation method, and resampled at 100‐ms intervals for Fast Fourier Transform (FFT) processing.

To clarify temporal relationships among changes in the RRI and the powers of LF and HF components, we used the UST spectral analysis using Fast Fourier Transform (FFT) with 5‐ and 25‐s time windows. In most cases, the time window was set to 25 s to make the frequency resolution 0.04 Hz, which is the lower frequency limit of the LF component. Furthermore, to detect the onset of changes in the HF power with even higher temporal resolution, the window length was occasionally set to 5 s, resulting in a frequency resolution of 0.2 Hz.

Before FFT processing, Hann window processing was performed. Based on the power spectrum of each time window, the LF and HF powers were calculated by integrating from 0.04 to 0.15 Hz and from 0.15 to 0.4 Hz, respectively.

**FIGURE 1:**
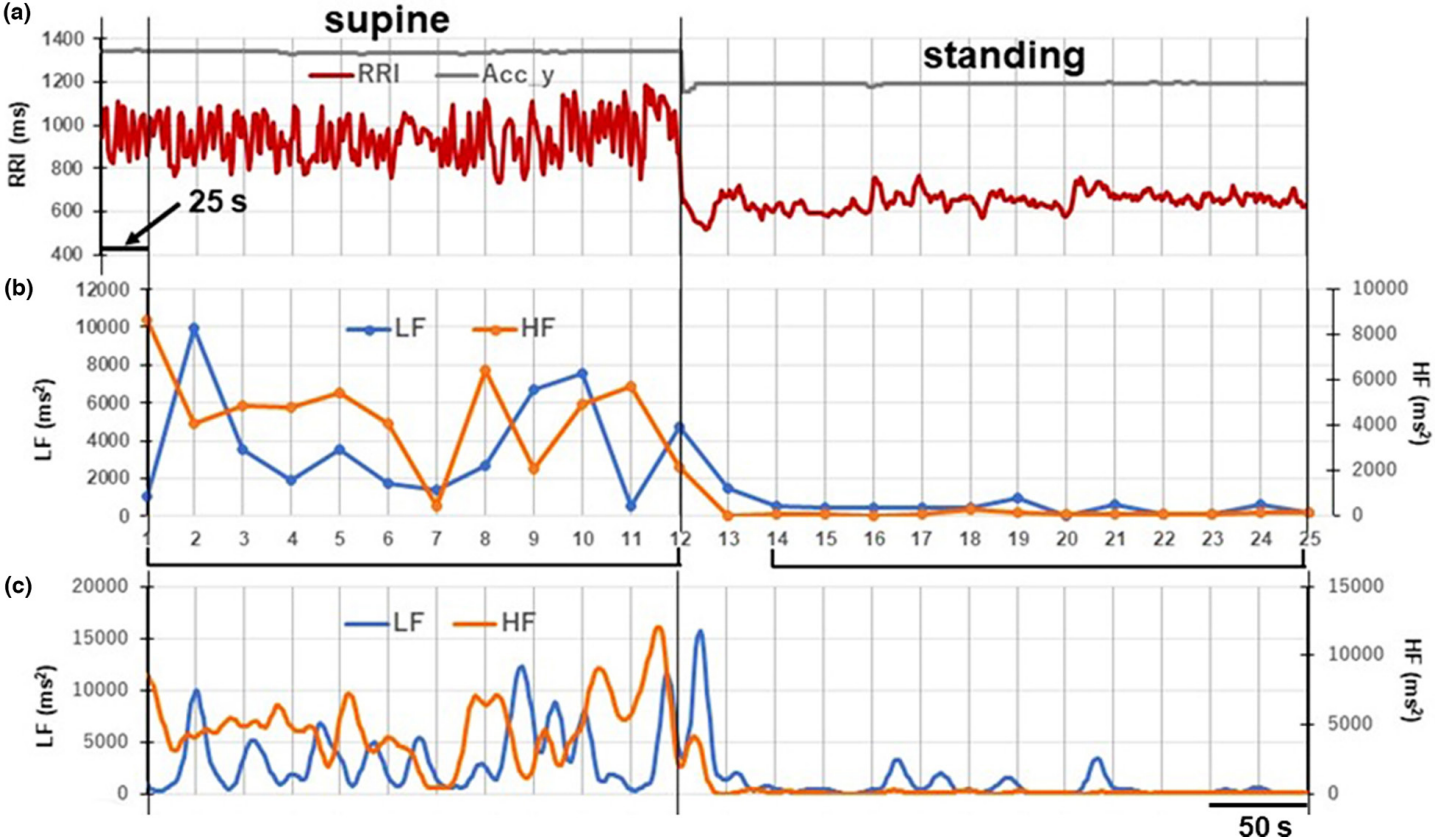
Example of changes in powers of low‐frequency (LF) and high‐frequency (HF) components of heart rate variability (HRV) that occurred upon standing from the supine position. (a) Effects of postural change from the supine position to standing on RRIs. The gray line (Acc_y) indicates a change in acceleration value in the vertical direction of the body axis that detects the time point of the postural change. The dark red line indicates the RRI time series. (b) Estimation of LF and HF powers in the supine position and during standing (25–25 analysis). RRI time series of 300 and 325 s were divided into 12 and 13 segments with 25‐s window lengths in the supine position and during standing, respectively, and the values of LF and HF powers obtained from each segment were plotted at the end of the segments as blue and orange dots, respectively. The means of LF and HF powers of segments numbered 1–12 on the *x*‐axis, and those numbered 14–25 were taken as the values of LF and HF powers in the supine position and during standing, respectively. The value obtained from the segment immediately after postural change (numbered 13) was omitted from the calculation of the mean. (c) Tracking the time course of changes in the LF and HF powers by performing a spectral analysis of 25‐s segments with 1‐s shift (1–25 analysis).

Figure 1 shows an example of the analyses of the RRI time series (Figure 1a) to examine changes in LF and HF powers over the supine to standing postural change. To assess the standing‐induced amplitude changes in LF and HF powers, the records of 300‐s and 325‐s before and during standing were divided into twelve and thirteen 25 s segments, respectively, and LF and HF powers were obtained in each 25‐s window length segment, plotted at the end of the segment (Figure 1b). The value obtained in the first segment immediately after the postural change was omitted from subsequent processing as it has been shown that, on active standing, the RRI decreases abruptly to a nadir and it takes ~20–30 s to reach a steady level of the decrease (Harms et al., 2021; Tanaka et al., 1996). The average value in each of the 12 segments, before and during standing, was then calculated. The ratios of these averages (standing:supine ratios of the mean LF and HF powers) were used as indices of change in the LF and HF powers associated with postural change. In the case shown in Figure 1, the means of LF and HF powers in 12 segments before standing were 3770.0 and 4484.2 ms^2^, while those during standing were 416.4 and 113.1 ms^2^, thus the LF and HF powers were reduced to 11% and 3%, respectively, upon standing.

To follow the time course of the amplitude changes in LF and HF powers continuously, a spectral analysis of the 25‐s segment was performed consecutively with a 1‐s shift, and each value thus obtained with a 1‐s interval was connected in Figure 1c. For convenience, the analyses shown in Figure 1b,c are subsequently referred to as the 25 (shift interval of the consecutive spectral analysis)‐25 (window length) and 1–25 analyses, respectively.

To estimate the temporal relationships among changes in the RRI and the LF and HF powers quantitatively, the cross‐correlations were calculated by the following equation:

$$C_{\mathrm{fg}}\left(\tau \right)=\frac{1}{L-\left|\tau \right|}\sum_{i=\max\left(0,\;-\tau \right)}^{\min\left(L-\tau ,\;L\right)}\left\{f\left(i+\tau \right)-\bar{f}\right\}\left\{g\left(i\right)-\bar{g}\right\}$$

where $f\left(i\right)$ and $g\left(i\right)$ are the pairs selected from the time series of RRI, LF and HF powers and time is denoted by i. L is the end time of the measurement. $\bar{f}$ and $\bar{g}$ are the averages of $f\left(i\right)$ and $g\left(i\right)$, respectively, so that the average values of $\left\{f\left(i+\tau \right)-\bar{f}\right\}$ and $\left\{g\left(i\right)-\bar{g}\right\}$ become zero.

### 2.5 Statistical analysis

The data are presented as the mean ± standard deviation (SD). However, when numerical data obtained by analyses showed a marked deviation from the normal distribution, they are presented using the median and interquartile range (IQR). Statistical comparison between two groups was performed by Mann–Whitney's *U* test.

## 3 RESULTS

### 3.1 Changes in LF and HF powers induced by postural change

**FIGURE 2:**
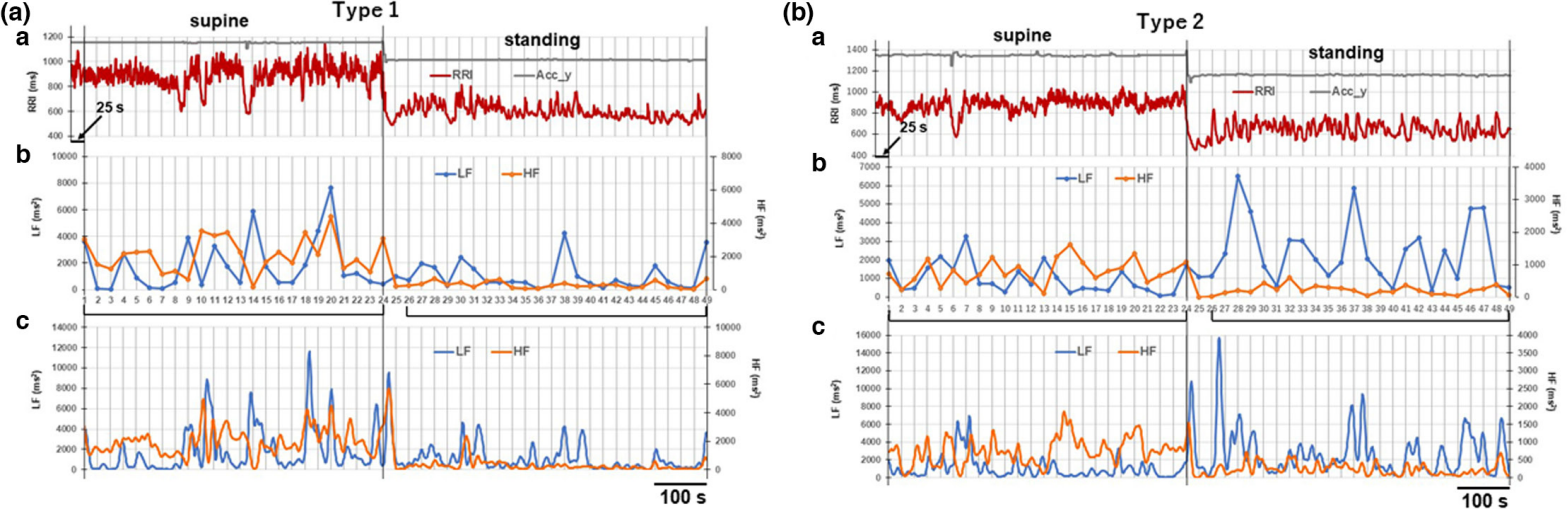
Two types of changes in LF powers that occurred upon standing from the supine position. (a) Example of a case in which LF powers were reduced during standing (type 1). (b) Example of a case in which LF powers were increased during standing (type 2). The values of LF and HF powers in the supine position and during standing were obtained as the averages of 24 segments of 25‐s window lengths in the supine position (numbered 1–24 on the *x*‐axis in Figure 2a[b],b[b]) and during standing (numbered 26–49), respectively. Note that in both cases HF powers were reduced by standing. (a[c],b[c]) Tracking the time course of changes in the LF and HF powers by performing a spectral analysis of 25‐s segments with 1‐s shift (1–25 analysis).

Figure 2 shows the effect of switching from supine to active standing on LF and HF powers. Subjects were asked to adopt the supine position for 15 min, and then to stand for 15 min. The RRI records for 600 and 625 s before and during standing were divided into twenty‐four and twenty‐five 25‐s segments, respectively, and the LF and HF powers were obtained by spectral analysis of the 25‐s window length of each segment. The mean LF and HF power values for each of the 24 segments, before and during standing, were calculated, and the ratio of the mean values while standing relative to that in the supine position (standing:supine ratios) was used as the index to estimate changes associated with standing. When calculating the average during standing, the value obtained from the segment immediately after standing was omitted for reasons described in the Methods.

As shown in Figure 2a,b, two patterns of change induced by standing were identified among 63 measurements from 20 subjects (3 times in 19 subjects, and 6 times in 1 subject). The quantitative data obtained from Figure 2 are shown in Table 1. In both Figure 2a,b, during standing the HF power invariably decreased with a marked decrease in the magnitude of the respiratory sinus arrhythmia (RSA) (Berntson et al., 1993; Eckberg, 1983; Katona & Jih, 1975; Taylor et al., 1999). In contrast, the LF power decreased in the case shown in Figure 2a, but increased in Figure 2b. We subsequently referred to the pattern when the standing:supine ratio of the LF power was ≤1 as type 1, and when the ratio was >1 as type 2. Among the 63 measurements, the former was detected in 49 cases in 20 subjects, and the latter detected in 14 cases in 8 subjects. In these 8 subjects, both type 1 and type 2 changes were detected.

**TABLE 1:** Two types of standing‐induced changes in high‐frequency (HF) and low‐frequency (LF) powers and R‐R interval (RRI)

	HF power (ms^2^)	LF power (ms^2^)	RRI (ms)
A Type 1 in Figure 2a			
Supine	2020.7	2046.8	903.2
Standing	471.7	1156.7	591.5
Ratio (standing/supine)	0.23	0.57	0.65
B Type 2 in Figure 2b			
Supine	793.6	1009.9	881.2
Standing	217.6	2418.0	645.1
Ratio (standing/supine)	0.27	2.39	0.73

**FIGURE 3:**
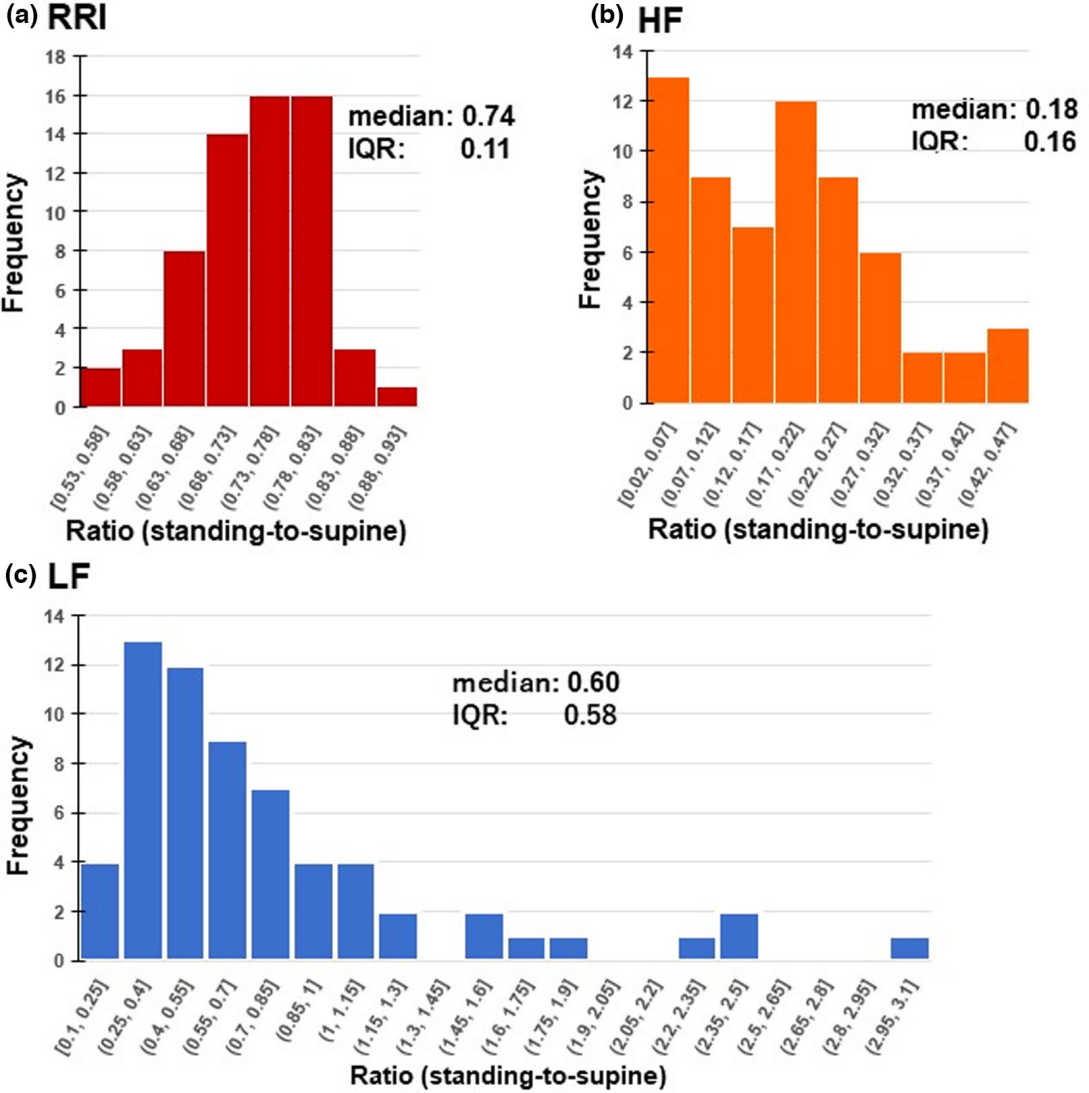
Histograms showing the distributions of the standing:supine ratios of RRI (a), HF power (b), and LF power (c) in total 63 measurements.

Figure 3 shows the distribution histograms of the standing:supine ratios of the RRI, and the HF and LF powers in the total 63 measurements. The ratio of HF power ranged from 0.03 to 0.47 (median 0.18, IQR 0.16), whereas that of LF power was widely distributed between 0.11 and 3.08 (median 0.60, IQR 0.58). The large variability in LF power change upon standing was consistent with a previous report in which an increase in LF power with head‐up tilting was observed in one‐third of healthy subjects, no significant change in one‐third, and a decrease in the remaining third (Hayano & Yuda, 2019).

It should be noted that while the ratio of the LF power was widely distributed, the HF power was invariably reduced by standing regardless of type 1 or type 2 case. The medians of the ratios of HF powers were 0.17 (IQR 0.15, *n* = 49) and 0.24 (IQR 0.15, *n* = 14) in type 1 and type 2 cases, respectively. Although this ratio tended to be slightly larger in type 2 cases, there was no difference between the two cases at the *p* < 0.05 level (*p* = 0.098, Mann–Whitney U test).

### 3.2 Temporal relationships among transient changes in the RRI and the LF and HF powers

**FIGURE 4:**
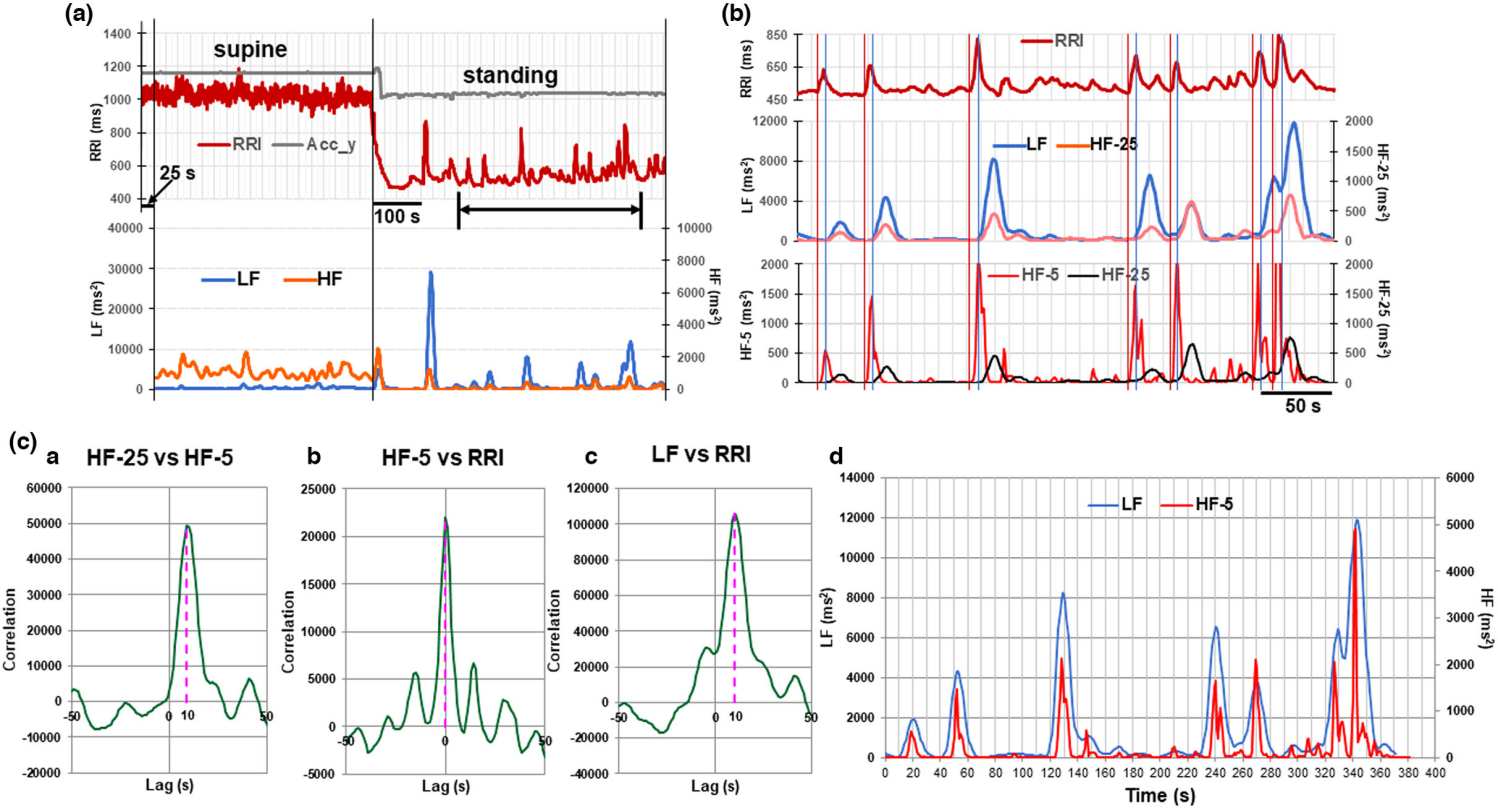
Temporal relationships among transient increases in the RRI and LF and HF powers during standing. (a) A typical example of a type 2 case. The postural change from the supine position to standing increased the frequency of the transient LF power increases. Although HF fluctuations were markedly suppressed during standing, small phasic increases in HF power were synchronized with the transient LF power increases. The portion indicated by the line with arrows at both ends is enlarged in Figure 4b. (b) (top and middle) temporal relationships among transient increases in the RRI and the LF and HF powers. Blue vertical lines were drawn through the starting points of the LF and HF power increases obtained by 1–25 analyses. (bottom) temporal relationship between transient increases in the RRI and HF powers. In this panel the time course of changes in HF power obtained by both 1–5 and 1–25 analyses were drawn in red (HF‐5) and black (HF‐25), respectively, for ease of comparison in the time course between them. Red vertical lines were drawn through the starting points of the transient RRI increases. These lines also went through the starting points of the HF‐5. (c) Results of cross‐correlation analysis. (c[a]) cross‐correlogram (CCG) for the pair of the HF‐25 and HF‐5 power increases. The CCG peak was detected at a lag of +10 s for the HF‐25 power increase. (c[b]) CCG for the pair of the HF‐5 power increase and the RRI. The CCG peak was detected at lag zero. (c[c]) CCG for the pair of the LF power increase and the RRI. The CCG peak was detected at a lag of +10 s for the LF power increase. (c[d]) superposition of the sequence of the LF power increase shifted to the left by 10 s on that of the HF‐5 power increase.

In type 2 cases, prominent increases in the LF powers that synchronized with the transient increase in the RRI, namely transient bradycardia, occurred frequently during standing. As high‐frequency fluctuations of the RRI and HF powers, due to RSA, were reduced during standing, type 2 cases provided a good opportunity to analyze the temporal relationship between transient changes in the RRI and LF powers. A typical example is shown in Figure 4a, where high‐frequency fluctuations of the RRI and HF power in the supine position were markedly suppressed during standing. To examine the temporal relationship between the increases in the RRI and LF power more precisely, the portion indicated by the line with arrows in Figure 4a is enlarged in Figure 4b. In the top and middle panels of Figure 4b, the transient increases in the RRI were accompanied by prominent increases in LF power, the onset of which was around the peaks of RRI increase, indicated by blue vertical lines.

In addition, we noticed that the transient increases in LF power were always accompanied by small but clear increases in HF power (Figure 4a,b middle panel). As it is generally accepted that HF power reflects modulation of heart rate mediated by cardiac vagal activity, this transient increase in HF power might relate to transient RRI increase. However, like LF power increase, the HF power started later than the RRI increase onset, which seemed to be incompatible with the notion that the transient increase in RRI is mediated by an increase in the cardiac vagal activity. We consider that this apparent contradiction is due to the limitation of time resolution in the calculation of frequency domain data. In Figure S1 we examined the effect of reducing the time window for spectral analysis on the temporal relationship between changes in the RRI and HF powers induced by the postural change from standing to the supine position. Reducing the time window for spectral analysis from 300 s, used for the conventional short‐term HRV measurement, to 100, 25, and 5 s gradually improved the time resolution for detecting transient changes in HF power, and with a 5‐s window the HF power increase occurred almost simultaneously with the RRI increase. Based on this finding, we tried to estimate the time course of changes in HF power using the 5‐s window in Figure 4b bottom panel. We found a sharp transient increase in HF power that occurred almost simultaneously with the onset of the RRI increase, shown by red vertical lines, and peaked around the time point of the peak of the RRI increase (HF‐5 in Figure 4b bottom). In this figure, the HF power increase detected by the 1–25 analysis (HF‐25) was drawn in black to facilitate the comparison of the difference in the time course from that detected by the 1–5 analysis drawn in red.

The temporal relationships among the RRI increase and the LF and HF power increases shown in Figure 4b were further examined by calculating their cross‐correlation functions in Figure 4c. In the cross‐correlogram (CCG) of the HF‐25 and HF‐5 power increases, a peak was detected with +10 s lag for the HF‐25 power increase (Figure 4c[a]), and in the pair of HF‐5 power increase and RRI increase the peak was detected at lag 0 (Figure 4c[b]), corroborating the finding shown in Figure 4b by red vertical lines that both occurred almost simultaneously. In contrast, for the pair of LF power increase with RRI increase, the peak was detected at the lag +10 s for the LF power increase (Figure 4c[c]). At first glance, it seemed that the LF power increase was delayed by 10 s compared to the RRI increase. However, it is more likely that this apparent delay of the LF power increase is due to FFT time window of 25 s. According to the definition of the LF power component in the framework of the frequency domain methods for HRV analysis, the window length must be set to 25 s or more (Billman, 2011; Task Force of the European Society of Cardiology and the North American Society of Pacing and Electrophysiology, 1996), which sacrifices time resolution, causing the delay in detecting the onset of the LF power increase. Thus, the CCG peak observed at +10 s lag between the RRI and LF power strongly suggests that this lag is caused by the delay of detecting the LF power increase due to the FFT window being set to 25 s. In Figure 4c[d], we shifted the LF power increase time series to the left by 10 s and superimposed it on that of the HF‐5 power increase under the assumption that the LF power increase occurs 10 s earlier than the timing detected by the 25‐s time window calculation. The result shown in this figure fits very well with the notion that the LF and HF power increases occur simultaneously during the transient RRI increase.

**FIGURE 5:**
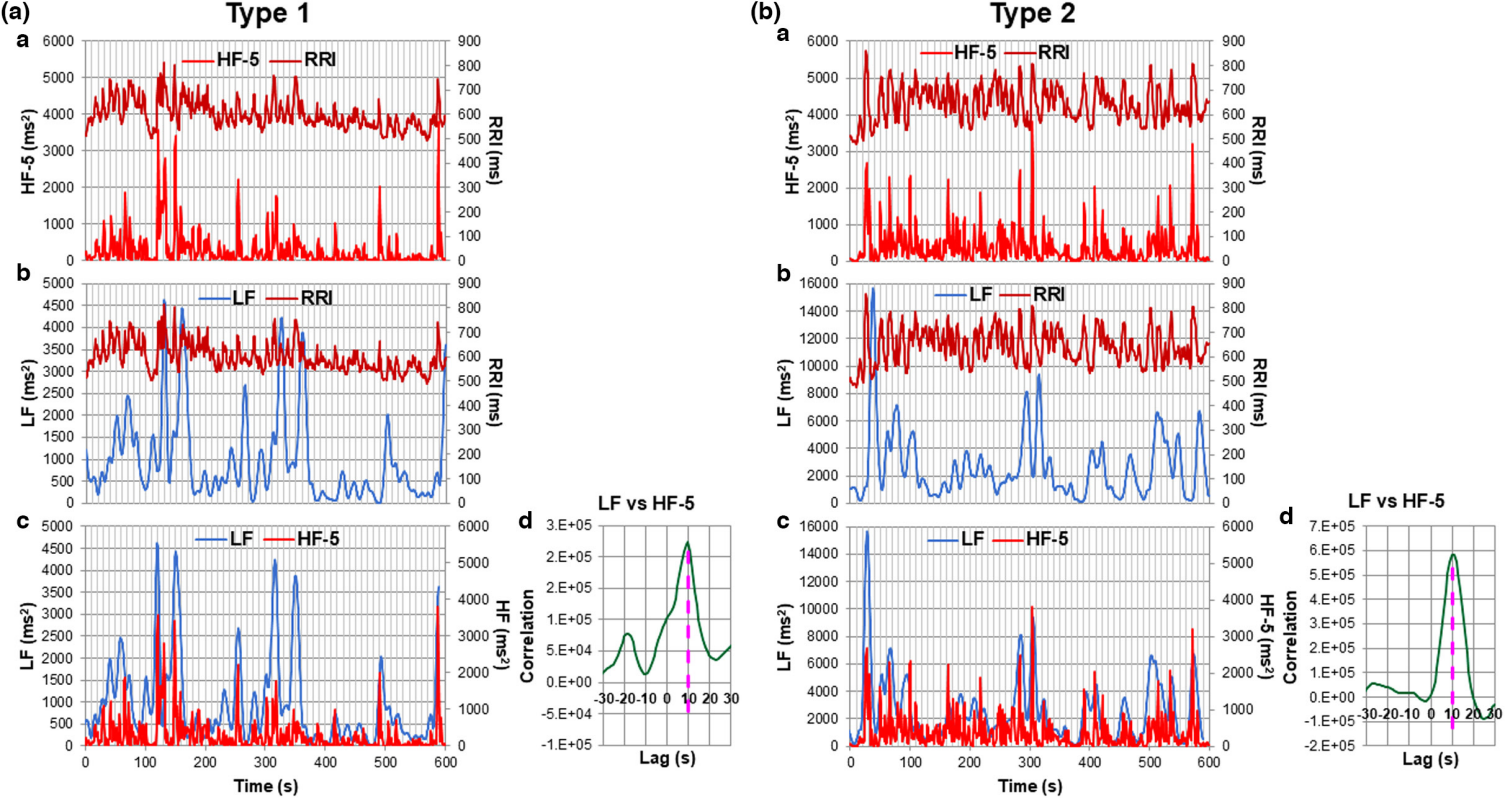
Temporal relationships among changes in the RRI and the LF and HF powers during 10 min standing in type 1 and type 2 representative cases. (a) Temporal relationship in the type 1 case. (a[a]) and (a[b]) RRI and HF‐5 power fluctuations in [a], and RRI and LF power fluctuations in [b] during 10 min standing. (a[c]) superposition of the sequence of the LF power increase shifted to the left by 10 s on that of the HF‐5 power increase. (a[d]) CCG for the pair of the LF and HF‐5 power fluctuations. The CCG peak was detected at a lag of +10 s for LF power fluctuation. (b) Temporal relationship in the type 2 case. The same analysis was performed as in (a).

In Figure 5, we estimated the temporal relationships among the RRI fluctuations and the LF and HF power increases for 10 min during standing by using the cross‐correlation analysis in the cases shown in Figure 2, which represented type 1 and type 2 cases. Panels [a] and [b] in Figure 5a,b show the temporal relationships among the RRI fluctuations and the HF‐5 and LF power increases for 10 min during standing in type 1 and type 2 cases, respectively. In the CCG between LF and the HF‐5 power increases, the peaks were detected at +10 s lag for the LF power increase in both cases (Figure 5a[d],b[d]). When the time series of the LF power increases were shifted to the left by 10 s and superimposed them on those of the HF‐5 power increases, the time series of the LF and the HF‐5 power increases were temporally well correlated in both type 1 and type 2 cases (Figure 5a[c],b[c]). Thus, we conclude that the LF and HF power increases occur simultaneously regardless of type 1 or type 2 cases during active standing.

An important issue was to examine whether the temporal relationships among the time series of the RRI increase, and LF and HF power increases during standing also held in the supine position. Unfortunately, due to RSA, the RRI and HF power fluctuations occurred much more frequently in the supine position, and therefore we were unable to temporally match most of them with the occurrence of the LF power changes which had much slower time courses. Thus, this issue was not resolved in this study.

## 4 DISCUSSION

In this study, we aimed to clarify the long‐controversial issues of origin and functional correlates of the LF component of HRV. As RRI fluctuates rapidly, it is obvious that the conventional short‐term power spectral density (PSD) analysis, typically for 5‐min segments of the time series, is not sufficient to solve this issue due to poor time resolution, as shown in Figure S1. To improve the time resolution for detecting the temporal relationship between RRI and LF power fluctuations, we set the time window for FFT to 25 s to make frequency resolution 0.04 Hz, which is the lower frequency limit of the LF component. Using this UST spectral analysis, we found that changes in the LF and HF powers occurred in association with both tonic and phasic changes in the RRI. We confirmed that, in all cases examined, the HF power fluctuations due to RSA were tonically suppressed during active standing. This suppression is considered to primarily result from sustained unloading of the arterial baroreceptors due to an upright posture which tonically diminishes baroreflex driving on vagal motoneurons (Berntson et al., 1993; Harms et al., 2021). On the other hand, the LF and HF powers increased phasically in association with the transient bradycardia (the transient RRI increase), probably via arterial baroreflex to blood pressure fluctuations (Cevese et al., 2001; Grasso et al., 1997). The amplitude of this phasic LF power increase was so large that the standing:supine ratio of the LF power was determined by the frequency of this event. When transient bradycardia occurred more frequently during standing than in the supine position, the standing:supine ratio of the LF power became >1, designated as type 2, with the opposite case designated as type 1. The type 2 case provided a good opportunity to examine the temporal relationship between transient changes in the RRI and the LF powers during standing, where high‐frequency fluctuations of the RRI and the HF powers due to RSA were markedly suppressed.

As the transient LF increase associated with the transient RRI increase was closely linked to the modest but clear HF power increase, we first analyzed the temporal relationship between the transient RRI and HF power increases during standing. It is accepted that the cardiac vagal activity is a major contributor to the HF component, supported by many experimental observations through autonomic maneuvers including vagotomy and muscarinic receptor blockade (Task Force of the European Society of Cardiology and the North American Society of Pacing and Electrophysiology, 1996). At first glance, however, our observation shown in the top and middle panels of Figure 4b seems to be incompatible with the above notion in that the HF power increase occurred later than the onset of the RRI increase. To settle this issue, we attempted to improve the time resolution for detecting the onset of the HF power increase by shortening the time window for FFT to 5 s and showed that the RRI and the HF power increase occurred almost simultaneously. This result reinforces the generally accepted notion that the HF power increase is mediated by enhanced cardiac vagal activity.

Concerning the temporal relationship between the LF and HF power increases that synchronized with the transient RRI increase during standing, we speculated that the apparent delay in LF power increase was due to setting the FFT time window to 25 s according to the definition of the LF power component in the framework of the frequency domain methods for HRV analysis. Considering this technical constraint we concluded that the LF and HF power increases occur simultaneously. The close link between changes in the LF and HF powers is supported by pharmacological experiments in which large‐dose atropine almost completely abolishes both components (Eckberg, 1997; Jokkel et al., 1995; Koh et al., 1994; Pomeranz et al., 1985).

Grasso et al. proposed that the LF power fluctuation in HRV is almost entirely accounted for by the baroreceptor reflex, as it is not generated in the absence of the LF fluctuation in arterial pressure (Cevese et al., 2001; Grasso et al., 1997). In their experiments, a cross spectral analysis showed high coherence between the RRI and systolic blood pressure in the LF range in eight healthy subjects in the supine position (Cevese et al., 2001). When the subjects were given the alpha‐adrenergic blocker (urapidil) to inhibit fluctuations in arterial pressure along with angiotensin II to maintain the mean blood pressure at control levels, LF power fluctuations in HRV were almost completely abolished in five subjects, and in the remaining three subjects, they were substantially reduced and lost their cross‐spectral characteristics with fluctuations of systolic blood pressure (Cevese et al., 2001).

Several lines of evidence indicating the close relationship between the LF power in HRV and baroreflex modulation of autonomic outflows have also been presented. Firstly, carotid sinus stimulation produced by neck suction increases the LF power in HRV only in individuals with normal baroreflex function (Sleigh et al., 1995). Secondly, the log of the LF power correlates positively with the log of the baroreflex‐cardiovagal gain, and chronic autonomic failure patients with low baroreflex‐cardiovagal gain have a low LF power (Moak et al., 2007; Rahman et al., 2011). Thirdly, carotid sinus, aortic, and combined sino‐aortic baroreceptor denervation decrease both the LF and HF powers in conscious mice (Rodrigues et al., 2011). These results indicate that the baroreflex plays a critical role in generating the LF power fluctuations in HRV. Based on these findings, we speculate the mechanism underlying the genesis of the transient increase in the LF power found in this study as follows; A transient rise in arterial pressure occurs sporadically due to naturally occurring physiological perturbations, which causes transient activation of the efferent vagal nerve and transiently decreases the heart rate through the arterial baroreflex. Increases in the LF and HF powers occur simultaneously in association with this transient activation of cardiac vagal activity. It must be confirmed in a future experiment that the suppression of the LF component by alpha‐adrenergic blockade in the supine position reported by Cevese et al. (2001) is also the case for the LF component found during active standing in this study.

We could not detect a statistically significant difference in the decrease in the standing:supine ratio of the HF power between type 1 and type 2 cases despite the link between transient LF and HF power increases during standing. This is most likely due to the modest contribution of the transient HF power increase associated with the LF power increase (see Figures 2b and 4) compared to that of the tonic decrease in HF power during the sustained standing position. However, mechanism(s) other than unloading of the arterial baroreceptors may be involved in the standing‐induced tonic decrease in HF power. It has been proposed that sympathetic nerve activity caused by orthostatic stress restrains vagally mediated RSA (Cohen & Taylor, 2002; Hedman et al., 1995; Taylor et al., 2001). Taylor et al. (2001) have shown that suppression of RSA by sympathetic activation on the sinoatrial node is substantial in young healthy subjects under 40^0^ head‐up tilt. They estimated that sympathetic nerve outflow caused by this mild orthostatic stress reduced RSA by ~56% during standard frequency (0.25 Hz) and normal tidal breathing. Although no conclusion has yet been reached as to mechanism(s) underlying these sympathetic influences, they would decrease the HF power equally both in type 1 and type 2 cases and obscure differences in the degree of the standing‐induced decrease in the HF power between the two cases.

### 4.1 Limitations

We were unable to determine whether the temporal relationships among changes in the RRI, and the LF and HF powers found during standing also applies to that in the supine position. High‐frequency fluctuations of RRI and HF power due to RSA in the supine position hindered the solution of this issue. We occasionally observed a prominent LF power increase that seemed to be associated with a sharp rise in RRI and HF power increase in the supine position. In most cases, however, we were unable to specify the temporal relationships of the LF power increases with the RRI and HF power increases that occurred much more frequently. To approach this issue, we may need to distinguish HF components of different origins. Cevese et al. (2001) reported that in the supine position, 45% of the RRI HF power fluctuation was preserved after the LF power fluctuation had been almost entirely suppressed by alpha‐adrenergic blockade. This indicates that nearly half of the HF power is independent of LF power, thus there are at least two types of HF components, connected with and unconnected to LF component, in the supine position. In a future experiment, it is needed to clarify differences in the dynamic properties and functional correlates between these two types of HF components.

In this study, the physiological basis for the large variability in LF power change upon standing remains unknown. The type 2 pattern (the standing:supine ratio of the LF power >1) occurred in the minority of subjects (8 out of 20 subjects). Furthermore, it was not always detected in these subjects, being detected in 14 cases in 27 measurements. These observations indicate that the type 2 pattern occurs in certain groups of subjects under certain conditions. To clarify the genesis of these inter‐ and intra‐subject variabilities we need to collect more data about in which specific groups of subjects and under what specific conditions the type 2 pattern is detected. Regarding the inter‐subject variability, differences in the standing:supine ratio of the LF power should be measured in different groups of subjects, such as subjects of different ages, trained athletes, or patients with baroreflex failure (Goldstein et al., 2011). Regarding the intra‐subject variability, the repeated measurements in the same subject are required to specify the conditions under which type 1 or type 2 pattern occurs.

## 5 CONCLUSION

The temporal relationships among RRI and transient increases in LF and HF powers of HRV were explored by UST spectral analysis. We found that during active standing, there were sporadic, transient RRI increases. The UST spectral analysis revealed that this RRI increase was associated with a simultaneous increase in HF power, which was also closely linked to the transient LF power increase. The results indicate that during standing, the increases in both LF and HF powers may reflect enhanced cardiac vagal activity which mediates the transient bradycardia.

## AUTHOR CONTRIBUTIONS.

S.O., and Y.Y. designed the study. S.O., Y.Y., H.N., C.U., and K.O. performed experiments. S.O., T.N., and S.M. analyzed the data. S.O. drafted the manuscript; T.N. S.M., and K.K. edited and revised the manuscript.

## FUNDING INFORMATION

This research did not receive any specific grants from funding agencies in the public, commercial, or not‐for‐profit section.

## CONFLICT OF INTEREST

The authors declare no conflicts of interest in association with the present study.

## ETHICS STATEMENT

This study was approved by the Ethical Committee of Takasaki University of Health and Welfare. All subjects provided written informed consents.

## Supporting information

Figure S1. Effect of reducing the time window for FFT on the time resolution for detecting increases in the HF powers induced by the postural change from standing to the supine position. A: Changes in RRI for 6 min active standing followed by 6 min supine position. The gray line (Acc_y) indicates a change in acceleration value in the vertical direction of the body axis that detects the time point of the postural change from standing to supine. B: Time courses of corresponding changes in the HF powers when the time window for FFT was reduced from 300 s (Ba: HF‐300) to 100 s (Bb: HF‐100), 25 s (Bc: HF‐25) and 5 s (Bd: HF‐5). Each time course of changes in the HF power was obtained by performing spectral analysis of 300‐, 100‐, 25‐ or 5‐s segments consecutively with a 1‐s shift. Note that as the time window was reduced from 300 s, which is used for the conventional short‐term HRV analysis, to 100, 25, and 5 s, the magnitude of the HF power fluctuation gradually increased and the latent time for detection of these changes became shorter. When the window was reduced to 5 s, the supine‐induced increase in the HF power was detected almost simultaneously with the occurrence of the RRI increase.
Figure S1
