# Supplementary figures and images for: Temporal relationships among changes in the RR‐interval and the powers of the low‐ and high‐frequency components of heart rate variability in normal subjects
Source: Physiol Rep. 2023 Jan 26;11(2):e15557. doi: 10.14814/phy2.15557 (PMC9879716; doi:10.14814/phy2.15557)

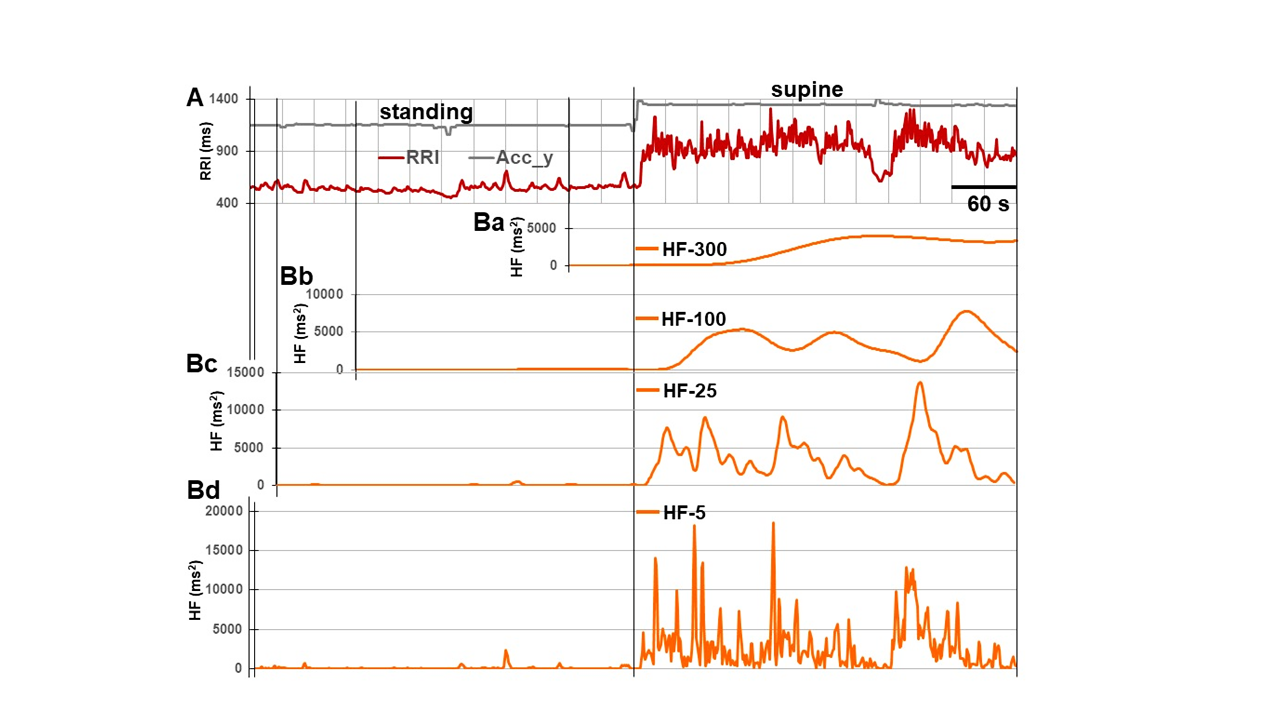

Supplement: Supplementary file 1 — Figure S1. Effect of reducing the time window for FFT on the time resolution for detecting increases in the HF powers induced by the postural change from standing to the supine position. A: Changes in RRI for 6 min active standing followed by 6 min supine position. The gray line (Acc_y) indicates a change in acceleration value in the vertical direction of the body axis that detects the time point of the postural change from standing to supine. B: Time courses of corresponding changes in the HF powers when the time window for FFT was reduced from 300 s (Ba: HF‐300) to 100 s (Bb: HF‐100), 25 s (Bc: HF‐25) and 5 s (Bd: HF‐5). Each time course of changes in the HF power was obtained by performing spectral analysis of 300‐, 100‐, 25‐ or 5‐s segments consecutively with a 1‐s shift. Note that as the time window was reduced from 300 s, which is used for the conventional short‐term HRV analysis, to 100, 25, and 5 s, the magnitude of the HF power fluctuation gradually increased and the latent time for detection of these changes became shorter. When the window was reduced to 5 s, the supine‐induced increase in the HF power was detected almost simultaneously with the occurrence of the RRI increase. Figure S1 [file PHY2-11-e15557-s001.tif]
